# An Evaluation of Otolaryngology Residency Program Websites

**DOI:** 10.7759/cureus.36231

**Published:** 2023-03-16

**Authors:** Alyssa Reese, Lauren DiNardo, Jeffrey Seeley, Tyler Le, Michele M Carr

**Affiliations:** 1 Otolaryngology, Jacobs School of Medicine and Biomedical Sciences, University at Buffalo, Buffalo, USA; 2 Otolaryngology, American University of the Caribbean, Cupecoy, MAF

**Keywords:** residency program website design, program website, residency match, residency application, residency training otolaryngology, otolaryngology residency

## Abstract

Introduction: Otolaryngology remains one of the most competitive specialties to match into during the residency application process. Medical students often apply to many programs to increase their chances of matching into a residency program and rely on residency websites to gather information about the programs they apply to. The purpose of this study was to determine the comprehensiveness of the information on otolaryngology residency program websites.

Methods: One hundred twenty-two publicly available otolaryngology residency program websites were evaluated for the presence of 47 criteria. Size, geographic location, and affiliation with a Top 50 ranked hospital for ear, nose, and throat care, according to the US News and World Report, were determined for each program. Frequencies were calculated for each of the different residency website criteria and non-parametric comparisons were used to analyze the relationship between the location, size, and ranking of each program, and the comprehensiveness of the program website.

Results: An average of 19.1 items (SD: 6.6 items) out of the 47 searches were present on the otolaryngology residency program websites. More than 75% of the websites contained the following program features: facility descriptions, descriptions of didactics, and research requirements. A total of 89.3% of the websites had a current resident list, 87.7% of these websites had photos of their residents, and 86.9% had a program contact email. Otolaryngology residency programs affiliated with a Top ENT hospital had a higher average number of criteria satisfied (21.6 criteria) compared to those that were not affiliated (17.9 criteria).

Conclusion: The inclusion of research selection criteria, call schedule/requirements, average Step 2 scores of matched residents, and social aspects of residency could improve otolaryngology residency applicant satisfaction with residency program websites. Updating otolaryngology residency websites will assist prospective applicants as they apply to a wide variety of residency programs.

## Introduction

​​The otolaryngology residency match is a highly competitive process, where fourth-year medical student applicants apply to a limited number of residency positions across the country. Almost every year, there are more applicants than the actual number of residency spots [1,2]. Due to the competitiveness of the otolaryngology MATCH, the number of applications per applicant to otolaryngology residency programs has risen astronomically. In 1992, the average otolaryngology applicant submitted 26 applications, an already high number of applications for the time. As of 2015, the average otolaryngology applicant submitted 64.5 applications, a nearly 250% increase over two decades. In 2021, the first virtual interview season, applicants applied to a mean of 72.7 otolaryngology residency programs [3].

Applying to more programs with the belief that this will lead to more interviews and a better chance to match has been labeled the “shotgun” approach [4]. However, this approach has drawbacks. Program interviewers must evaluate a large number of applications and applicants have an additional financial burden, spending $1000-13,225 on interviews depending on the specialty [5]. This requires many applicants to significantly increase their medical school debt [6].

In congruence with their desire to match in a highly competitive field, otolaryngology applicants will apply to a large number of programs, often due to an inability to definitively differentiate between programs. Many applicants utilize the internet when trying to compare residency programs. This implies that residency program websites are a key component in helping applicants decide where to apply. Therefore, the content of otolaryngology residency program websites is of great interest to applicants, with the potential to help them decide which programs may be their best fit. This study seeks to determine the current comprehensiveness of the information on otolaryngology residency program websites.

## Materials and methods

One hundred twenty-two publicly available otolaryngology residency program websites were evaluated for the presence of information that would be relevant to prospective otolaryngology residency program applicants in July 2021. The total list of 125 programs was accessed through the American Medical Association FREIDA (Fellowship and Residency Electronic Interactive Database). Two programs did not have a website linked to FREIDA and one program had no information on its webpage. After identifying the programs, four of the authors (AR, LD, TL, and JS) searched for a total of 47 different criteria on the websites. Criteria needed to be present directly on the page or accessible through a listed link or search tab on the program website. Links that were not directly on the program website or able to be found via the search engine were not accessed. These 47 criteria were determined based on the results of a prior study conducted by DiNardo et al. [7].

Both the size and the geographic location of the otolaryngology residency programs were classified. Program size refers to the number of otolaryngology residents accepted into each program per year. A program was classified as large if it accepted three or more residents per year and small if it accepted two or less. Programs that alternated between two and three residents per year were classified as small based on the mean number of residents per year being below three. The location of each program was categorized as Northeast, Midwest, West, and South based on the U.S. Census Bureau classification [8]. Lastly, using the US News and World Report, the top 50 ranked hospitals for ear, nose, and throat care that had an accredited otolaryngology residency program were grouped and compared to the programs that were not ranked [9]. A total of 48 of the 50 ranked hospitals had a corresponding residency program.

Frequencies were calculated for each of the different residency website criteria. Non-parametric comparisons were also utilized in order to analyze the relationship between the location, size, and ranking of each program, and the comprehensiveness of the program website. The methods of this study were created based on two prior studies that evaluated residency program websites [10,11].

## Results

Information on the residency program region, size, and whether the program was affiliated with a top 50 otolaryngology hospital can be found in Table 1. The mean number of residents per class was 3.0 with 76 (62.2%) of the programs being classified as large (>3 residents per year).

**Table 1:** Overall number of features present on otolaryngology residency program websites organized by hospital ranking, size of the program, and US Census region. *Based on the top 50 ranked hospitals for “ear, nose, and throat care,” with an accredited otolaryngology residency program, according to the US News and World Report. **Large programs were classified as three or more residents per year. Small programs were classified based on less than three residents per year.

Top ENT Hospital*	Average Number of Criteria Satisfied	Standard Deviation of Criteria Satisfied
Yes	21.6	4.8
No	17.9	6.8
Size of Program**		
Large	20.1	6.1
Small	17.8	6.7
Region		
Northeast	19.4	5.1
South	18.8	6.5
Midwest	19.5	6.18
West	19.2	8.4

Out of the 47 factors searched for on the websites, programs contained an average of 19.1 items (SD: 6.6 items). Table 2 shows the website features and their presence on the websites reviewed. Over 50% of the websites had the following application and employment features: link to Electronic Residency Application Service (ERAS), away rotation information, interview information, salary, benefits, and vacation time (Table 2). More than 75% of the websites contained the following program features: facility descriptions, descriptions of didactics, and research requirements (Table 2). One hundred and nine program websites (89.3%) had a current resident list and 107 of these websites (87.7%) had photos of their residents. One hundred and six program websites (86.9%) had a program contact email (Table 2). The overall number of features present on otolaryngology residency program websites is organized by hospital ranking, size of the program, and US Census region in Table 3.

**Table 2:** Information found on otolaryngology residency websites. *Top 10 website criteria sought after by applicants in DiNardo et al.

Website Criteria	Number of Websites with Criteria N (%)
Application Criteria	
Link to ERAS (Electronic Residency Application Service)	77 (63.1)
Step 1 Score Minimum Requirement	9 (7.4)
Average Step 1 Score of Current Residents	1 (0.8)
Step 2 Score Minimum Requirement	6 (4.9)
Average Step 2 Score of Current Residents	1 (0.8)
Research Selection Criteria*	4 (3.3)
Clerkship Grade Selection Criteria	2 (1.6)
Letters of Recommendation Requirements	48 (39.3)
Away Rotation Information	63 (51.6)
Interview Information	62 (50.8)
Employment Aspects	
Salary Information*	80 (65.6)
Benefits Information*	79 (64.8)
Parking Information	33 (27.0)
Meal Allowance Information	43 (35.2)
Vacation and Time Off Policy	63 (51.6)
Program Features	
Facility Descriptions	94 (77.0)
Description of Didactics	96 (78.7)
Research Requirements*	92 (75.4)
Active/Past Research Projects	55 (45.1)
Intern Year Schedule*	90 (73.8)
Rotation Schedule (Postgraduate years 2-5)*	85 (69.7)
Surgical Case/Responsibility Progression	32 (26.2)
Call Schedule/Requirements*	44 (36.1)
Resident Case Volumes	20 (16.4)
Resident Training Events*	90 (73.8)
Fellowship Programs Available at Institution	51 (41.8)
Arts/Humanities Activities in Residency	3 (2.5)
Virtual Access to Department	19 (15.6)
Program Video	62 (52.5)
Social/Community	
Family/Medical Emergency Policies	40 (32.8)
Child Birth Leave Policies	37 (30.3)
Child Care Information	10 (8.2)
International Mission Opportunities	24 (19.7)
Health and Wellness Support	51 (41.8)
Statement on Diversity, Equity, and Inclusion	31 (25.4)
Information About the City	70 (57.4)
Faculty Information	
Current Resident List*	109 (89.3)
Current Resident Photos*	107 (87.7)
Current Resident Email Addresses	12 (9.8)
Current Resident School History	98 (80.3)
Current Resident Bio	54 (44.3)
Career Placement of Past Residents	67 (54.9)
Message From the Chairperson	26 (21.3)
Message From the Program Director	48 (39.3)
Message From the Current/Past Residents	11 (9.0)
Program Contact Email*	106 (86.9)
Link to Program’s Social Media	38 (31.1)

**Table 3:** Otolaryngology residency program’s region, size, and top 50 rankings.

Residency Program Demographics	Number of Programs N (%)
Region	
Northeast	31 (25.4)
South	37 (30.3)
Midwest	33 (27.0)
West	21 (17.2)
Size of Program	
Small (<3 residents per year)	46 (37.7)
Large (>3 residents per year)	76 (62.3)
Top 50 Hospitals for ENT	
Yes	44 (36.1)
No	78 (63.9)
Average Number of Residents Per Year	3.0 residents

## Discussion

Otolaryngology residency program websites assist applicants in determining if a residency program is a good “fit” for them. These websites gained importance during the virtual interviews of the 2020-2021 application season, and continue to be important as otolaryngology programs offer virtual interviews. This study sought to evaluate the comprehensiveness of otolaryngology residency program websites, as residency programs continue to update their websites to support the virtual nature of residency program exploration during the COVID-19 pandemic.

This study expands upon an analysis of otolaryngology residency program websites that was conducted in 2014 [10]. Svider et al. evaluated each otolaryngology residency program website for 23 factors, compared to our study which determined the presence of 47 factors. The previous study also only evaluated 99 program websites that were available, compared to the 122 program websites in the present study. In the time since the present study was conducted, another similar study was conducted by Tong et al. [12]. Investigators looked at the presence of a variety of components on 118 otolaryngology residency websites between December 2020 and January 2021 [12]. The increased number of program websites is reflective of the increase in the social media presence of otolaryngology residency programs. In the last decade, the social media presence of otolaryngology residency programs has increased, with at least 61% of programs having at least one social media account [13].

The criteria evaluated in this study were determined based on a survey study conducted by DiNardo et al. that surveyed 101 fourth-year medical students applying to otolaryngology, re-applicants applying to otolaryngology, and current first-year otolaryngology residents [7]. A total of 38 criteria were listed and participants were asked to mark the components they hoped to see on otolaryngology residency websites. Ultimately, they found that the top 10 components were sought by at least 80% of participants. These included the current resident list with photos, intern year schedule, call schedule/requirements, rotation schedule, comprehensive faculty list, benefits information, research requirements, salary information, research selection criteria, program contact email, and specific extra courses for residents [7]. Frequencies of each component in the present study are indicated by an asterisk in Table 2. While most of the top 10 components were found on most otolaryngology residency websites, research selection criteria were only present on 3.3% of the websites, and call schedule requirements were only found on 33.1% of websites. Thus, there is a gap between what otolaryngology applicants wish to see on otolaryngology residency websites and what is present on these websites.

The United States Medical Licensing Examination (USMLE) Step 1 and Step 2 score minimum requirements were only listed on less than 10% of the websites and few websites contained average Step 1 and Step 2 scores of current residents [14]. The lack of this information contradicts the importance of these scores, as a survey of otolaryngology residency program directors found that prior to COVID-19, 37.9% believed that USMLE Step 1/COMLEX Level 1 score was one of the three most important criteria in selecting a candidate, compared to 41.4% for a COVID-19 affected cycle [15]. Thus, the lack of information regarding Step 1 and Step 2 scores demonstrates an area where the lack of this knowledge could lead to uninformed applications. Given that the Step 1 USMLE scores are now scored as pass or fail, the desire to see an average Step 2 score on residency websites may increase even further. Transparency with regard to mean scores would help applicants select programs that are within reach based on their Step 1 and Step 2 scores. This may help to reduce the number of applicants unlikely to match at specific programs, in turn reducing the number of applications needing review by program directors.

Svider et al. and Tong et al. did not evaluate any website components associated with social aspects of the prospective resident’s life, with the exception of a video highlighting the resident's life and photos of the resident's activities in Tong et al.’s study [10,12]. Our study sought to evaluate program websites for a variety of social criteria, including paternity/maternity leave policies, child-care, and family/medical emergency leave policies. In doing so, we found this area to be lacking, as child-care, childbirth leave policies, and family/medical emergency policies were found only on 8.2% (N=10), 30.3% (N=37), and 32.8% (N=40) of websites, respectively. Studies have found that female gender and lifestyle concerns are associated with the desire to leave surgical careers, and women that have children during residency may be at a greater risk of attrition [16-18]. Thus, the absence of this information may be discouraging to those who wish to have a child during residency. This information would allow applicants to make more informed decisions about which programs would suit their needs in the area of family planning. In turn, this may result in decreased attrition rates.

Despite the increase in advocacy for diversity in medicine that has occurred in the last decade, only 31 (25.4%) of the program websites contained a statement that reflected their stance on the diversity of their program or their commitment to diversity. These statistics corroborate the findings of Driesen et al., who sought to determine the presence of diversity elements on general surgery program websites [19]. One of the elements that they analyzed was the presence of a program-specific diversity and inclusion message, which was only contained by 19% of the 242 general surgery programs. The lack of these statements is concerning, as a recent study found that both women and non-white otolaryngology residency program applicants prioritized program diversity with gender and racially congruent faculty and resident mentors more than men and white applicants [20]. However, 107 (87.7%) of program websites did contain photos of the current residents. The diversity of the program’s residents may be judged by an applicant through these portraits.

While the comprehensiveness of otolaryngology residency program websites has increased since the 2014 study by Svider et al., many websites still lack components that candidates may deem useful when deciding where to apply and creating their rank list. A survey of prospective otolaryngology residency applicants in the 2020-2021 match found that 53 (62.4%) applicants did not believe they would gather sufficient information to make informed decisions about their own rank list [21]. Thus, an improvement of these websites may assist prospective applicants in making informed decisions about where they wish to apply, instead of using the “shot-gun” approach, especially with the presence of virtual interviews. Future research is needed in order to determine the utility of each website component for prospective applicants.

Limitations

Since each otolaryngology program website was very different, the aesthetic qualities and ease of use of the website were not quantified. Additionally, this study only looked at criteria present directly on the page, or accessible through a listed link or search tab on the program website page as of July 2021. Thus, there may have been criteria, such as salary and benefits, listed elsewhere on the hospital website that was not found and tallied. The program websites could have been updated between the time of analysis and the publication of this study. Overall, a major limitation was also the subjectivity in determining which criteria to include. However, the authors utilized DiNardo et al.’s study and attempted to be comprehensive in the criteria in order to diminish the effect of this limitation.

## Conclusions

With an increased focus on virtual interviews during the COVID-19 pandemic, otolaryngology residency programs should update their websites to include comprehensive criteria in order to attract prospective applicants. The presence of research selection criteria, call schedule/requirements, average Step 2 scores of matched residents, and social aspects of residency could improve otolaryngology residency applicant satisfaction regarding residency program websites. Updating these websites will assist prospective applicants in making informed decisions about where they wish to apply and thus, reduce the high costs of the residency application season.
